# Development of specific monoclonal antibodies for the detection of natural chicken tumor necrosis factor-alpha

**DOI:** 10.1016/j.heliyon.2022.e12446

**Published:** 2022-12-21

**Authors:** Yi Yang, Yining Meng, Lina Chen, Maoli Dong, Huining Zhang, Ji Wu, Xiaoli Hao, Shuangjiang He, Yunfei Tian, Zaicheng Gong, Shaobin Shang

**Affiliations:** a Institute of Comparative Medicine, College of Veterinary Medicine, Yangzhou University, Yangzhou 225009, China; b Jiangsu Co-Innovation Center for Prevention and Control of Important Animal Infectious Diseases and Zoonoses, Yangzhou University, Yangzhou 225009, China; c International Corporation Laboratory of Agriculture and Agricultural Products Safety, Yangzhou University, Yangzhou 225009, China

**Keywords:** chTNF-α, Monoclonal antibody, Polyclonal antibody, Epitope mapping, Antigen-capture ELISA

## Abstract

Tumor necrosis factor alpha (TNF-α) is an important proinflammatory cytokine and the only known cytokine that can directly kill tumor cells. Unlike mammalian counterparts, chicken TNF-α (chTNF-α) gene has not been identified until very recently due to its high GC content (∼70%) and long GC fragments. The biological functions of this newly-identified cytokine and its detection methods remain to be further investigated. In this study, the extracellular domain of chTNF-α was cloned into prokaryotic vector after codon optimization and recombinant chTNF-α protein was expressed. Subsequently, using recombinant chTNF-ɑ as immunogen, rabbit polyclonal antibody (pAb) and eight clones of mouse anti-chTNF-ɑ monoclonal antibodies (mAbs) were produced, respectively. Both the pAb and mAbs specifically recognized recombinant chTNF-ɑ expressed in *E.coli* and transfected COS-7 cells. Further mapping the antigenic region showed that all the mAbs recognized a region of amino acid residues 195–285 of chTNF-ɑ. Furthermore, an antigen-capture enzyme-linked immunosorbent assay for the detection of chTNF-ɑ was established using one mAb and the pAb. This assay showed no cross-reactivity with irrelevant Trx-fused antigens and could detect natural chTNF-ɑ expressed by mitogen-activated chicken splenocytes in a dose-dependent manner, with a detection limit of 1 ng/mL. Collectively, our results indicated that the mAbs and pAb against chTNF-α are specific and could be used for the study of the biological functions of chTNF-ɑ and the detection of chTNF-ɑ.

## 1 Introduction

Tumor necrosis factor alpha (TNF-α) is an important inflammatory cytokine mainly produced by activated monocytes and macrophages. It was initially described as a circulating factor that can cause the necrosis of tumors, and was subsequently identified to have a variety of biological activities and play roles in immune responses in mammals, such as killing and inhibiting tumor cells, promoting the phagocytosis of neutrophil, and anti-inflammation (Balkwill, 2006; Jang et al., 2021). However, as an endogenous pyrogen that causes fever, chronic exposure to a low dose of TNF can cause cachexia, wasting syndrome, and depression (Chu, 2013). TNF-α exists in two bioactive forms, namely transmembrane TNF-α (tmTNF-α) (∼26 kDa) and soluble TNF-α (sTNF-α) (∼17 kDa), respectively. tmTNF-α is the precursor of sTNF-α, and represents the extracellular domain of tmTNF-α. After cleaving by TNF-α-converting enzyme (TACE), sTNF-α can be released from the tmTNF-α and subsequently displays its biological functions by binding to TNF receptors (TNFR1 and TNFR2) (Miao et al., 2020).

The identification of novel avian cytokines and their receptors has been severely hampered due to a lack of diagnostic tools and their low homology with mammalian orthologs. This dilemma has been obviously improved when International Chicken Genome Sequencing Consortium (ICGSC) released the first draft whole genome sequence of *Gallus gallus* in 2004 (International Chicken Genome Sequencing, 2004) and an updated annotation recently. In the early 21st century, TNFR1 and TNFR2 were successively identified and characterized in avian species (Abdalla et al., 2004; Bridgham and Johnson, 2001, 2003). However, chTNF-α was not identified by that time though extensive searches of 60 avian genomes have been done. Therefore, it was believed for a long time that the cytokine of TNF-α might have been lost during the evolution of avian species (Lovell et al., 2014). Until very recently, Rohde et al. successfully cloned the entire chicken TNF-α (chTNF-α) coding sequence from Lohmann Selected Leghorn (LSL) chickens with overlapping PCR (Rohde et al., 2018). The coding sequence of *chTNF-α* gene composes of 858 nucleotides (nt), with 70% GC content, encoding a protein of 285 amino acids. The extracellular domain of chTNF-α (298–855 nt) showed 45% homology with human counterpart. Recombinant chTNF-α expressed in HEK 293 cells showed biological activity in activating NF-κB in CEC-NFκB-luciferase reporter cells through signaling transduction (Rohde et al., 2018).

To date, studies on avian inflammation have mainly focused on interleukins and chemokines, including IL-1, IL-6, IL-10 and TGF-β (Burggraaf et al., 2014; Dalgaard et al., 2015; Li et al., 2022; Schneider et al., 2001). The discovery of TNF-α in chicken has necessitated the detection and functional analysis of chTNF-α during disease and immunity in the future. In this study, we expressed the extracellular domain of chTNF-α in *E. coli*, generated rabbit polyclonal antibody (pAb) and mouse monoclonal antibodies (mAbs) against chTNF-α and determined the antigenic region recognized by the mAbs. In addition, using the pAb and one mAb, we developed and validated an antigen-capture enzyme-linked immunosorbent assay (ELISA) for the detection of natural chTNF-ɑ expressed by chicken immune cells.

## 2 Materials and methods

### 2.1 Stimulation and quantification of natural chTNF-ɑ

Peripheral blood mononuclear cells (PBMCs) were isolated from whole blood of a two-month-old specific-pathogen-free (SPF) White Leghorn chicken and cultured in a 24-well plate with three million cells per well. After overnight culture, the adherent macrophages were treated with lipopolysaccharide (LPS) (100 ng/mL) for 2, 4 and 8 h, respectively, and subsequently harvested for RNA extraction. The expression level of *chTNF-ɑ* was quantified using SYBR Green real-time PCR with forward primer: 5′-CGCTCAGAACGACGTCAA-3′ and reverse primer: 5′-GTCGTCCACACCAACGAG-3'.

### 2.2 Construction of recombinant chTNF-ɑ plasmids

**Figure 1:**
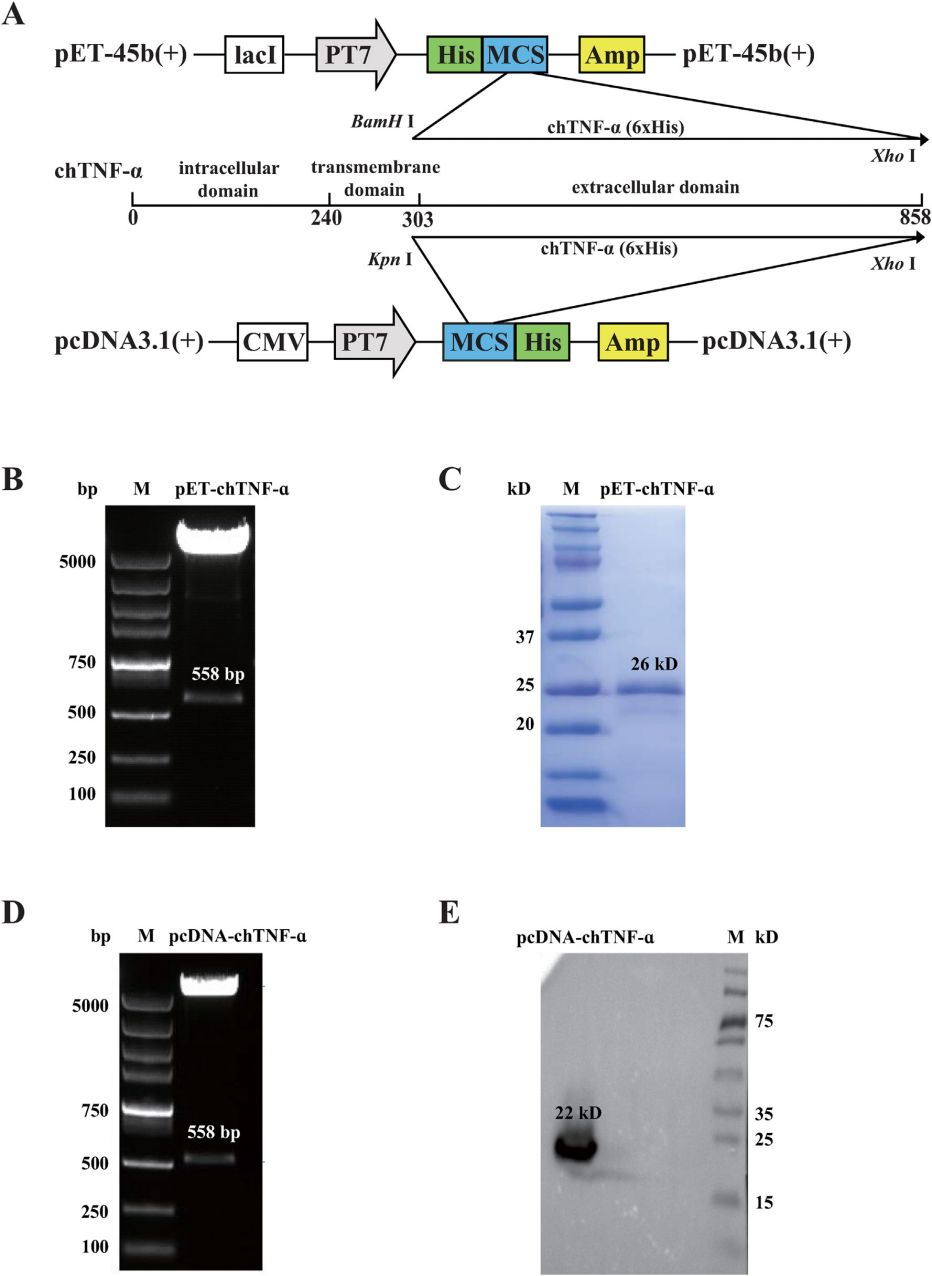
The construction of prokaryotic and eukaryotic recombinant vectors expressing chTNF-ɑ. (A) The codon optimized sequence of chTNF-ɑ extracellular domain (298–855 bp) was ligated into *BamH* Ⅰ-*Xho* Ⅰ site of pET-45b (+) vector and *Kpn* Ⅰ-*Xho* Ⅰ site of pcDNA3.1 (+) vector. (B) The identification of pET-chTNF-ɑ by double digests. (C) The identification of prokaryotic recombinant chTNF-ɑ. (D) The construction of pcDNA-chTNF-ɑ. (E) The identification of pcDNA-chTNF-ɑ by double digests. (F) The identification of eukaryotic recombinant chTNF-ɑ. Primary antibody: mouse anti-His Tag monoclonal antibody (Jackson ImmunoResearch, West Grove, USA). Second antibody: HRP-labeled goat anti-mouse IgG (H + L) polyclonal antibody (Jackson ImmunoResearch, West Grove, USA). The uncropped images of Figure 1 (B–E) were referred in Supplementary Figure 5.

The nucleotide sequence of extracellular domain of *chTNF-ɑ* (298–855 bp) was obtained from GenBank accession number MF000729 and synthesized by a commercial biological laboratory (HuaBio, Hangzhou, China) after codon optimization. Plasmids pET-45b and pcDNA3.1 (+) were used in the construction of prokaryotic and eukaryotic recombinant vectors expressing chTNF-ɑ. For the construction of prokaryotic vector, the cDNA encoding *chTNF-ɑ* was cloned and ligated into the pET-45b bacterial expression vector fused with a His-tag (pET-chTNF-ɑ) using the following primers: 5′-CGCGGATCCGAAGATCCGACCGCCCCGC-3′ and 5′-CCGCTCGAG (6xHis)ATGATCAACGCCAACAACACCAAAATAGGCAC-3'. Similarly, for the construction of eukaryotic vector, the cDNA encoding chTNF-ɑ was cloned and ligated into the pcDNA3.1 (+) cellular expression vector (pcDNA-chTNF-ɑ) containing a His-tag with a forward primer: 5′-CGCGGTACCATGGCCACCGAAGATCCGACCGCCCCGC-3′ and the same reverse primer as the prokaryotic vector (Figure 1A). Recombinant vector pET-chTNF-ɑ was transformed into BL21 (DE3) competent cells (Takara, Kyoto-shi, Japan) and induced with 1.0 mM Isopropyl-beta-D-thiogalactopyranoside (IPTG) (Sigma-Aldrich, St. Louis, USA) for 4 h at 37 °C. Then the cultures (300 mL) were centrifuged at 18,000 x g for 10 min, and the cell pellets were resuspended in 15.0 mL of 10 mM phosphate buffered saline (PBS) (Solarbio, Beijing, China). The recombinant chTNF-ɑ was purified using Ni-NTA agarose (GE Healthcare, Chicago, USA) and Imidazole solutions (10 mM, 30 mM, 60 mM, 400 mM, 500 mM) (Beyotime, Shanghai, China). The concentration of purified chTNF-ɑ protein was determined using Bicinchoninic Acid (BCA) assay, and the purity was confirmed by sodium dodecylsulfate-polyacrylamide gel electrophoresis (SDS-PAGE) and Western blot analysis.

### 2.3 Production and characterization of polyclonal and monoclonal antibodies against chTNF-ɑ

The recombinant chTNF-ɑ from *E. coli* was used as an immunogen to generate anti-chTNF-ɑ polyclonal and monoclonal antibodies from rabbits and mice, respectively. Two New Zealand white rabbits (male, ∼2.5 kg) were immunized at one-week interval for three times by multiple subcutaneous injections with 200 μg of recombinant chTNF-ɑ mixed with complete (for the first immunization) or incomplete (for boosters) Freund's adjuvant (Sigma-Aldrich, St. Louis, USA). At seven days after third booster immunization, blood samples were collected from the marginal vein of the rabbits' ears. After centrifuged at 2,000 x g for 10 min at 4 °C, the titers of polyclonal antibodies were determined by ELISA.

For the generation of mAbs, three BALB/c mice (female, 6–8 weeks) were immunized every two weeks by intraperitoneal and subcutaneous injections with 50 μg of recombinant chTNF-ɑ emulsified with complete (for the first immunization) or incomplete (for boosters) Freund's adjuvant (Sigma-Aldrich, St. Louis, USA). The titers of polyclonal antibodies were determined by ELISA at seven days after third booster immunization. An additional booster immunization was performed when the titer was above 1:12,800. Three days later, mice were euthanized and the spleens were obtained aseptically. Then splenocytes were fused with mouse myeloma cell line SP2/0 at the ratio of 10:1 with polyethylene glycol (PEG) (Thermo-Fisher Scientific, Waltham, USA) according to standard procedures. The fused cells were cultured continually with RPMI 1640 medium containing hypoxanthine-aminoprerin-thymidine (HAT). Resulting hybridoma colonies were maintained on RPMI 1640 medium containing hypoxanthine-thymidine (HT). Hydridoma supernatants were screened by Western blot, Enzyme linked immunosorbent assay (ELISA) and indirect immunofluorescence for the presence of chTNF-ɑ-specific antibodies.

### 2.4 Indirect ELISA

Briefly, 96-well microtiter plate was coated with 100 μL of the purified chTNF-ɑ (1.0 μg/mL) overnight at 4 °C, followed by blocking with 5% skim milk for 2 h at 37 °C. After blocking and thoroughly washing, the plates were incubated at 37 °C for 1 h with 100 μL of polyclonal serum/hybridoma culture supernatant and washed five times with PBST. Horseradish peroxidase (HRP)-labeled goat anti-rabbit or mouse IgG monoclonal Ab (1/10,000 dilution in 2% fetal bovine serum PBS) (Thermo-Fisher Scientific, Waltham, USA) was added and incubated at 37 °C for 45 min. After washing, color was developed with 100 μL of 3,3′,5,5′-tetramethylbenzidine (TMB) substrate in dark at room temperature. Optical density (OD) was measured at 450 nm with an automatic microplate reader (TECAN, Männedorf, Switzerland).

### 2.5 Isotyping of immunoglobulins

A commercial ELISA kit (BD Biosciences, San Jose, USA) containing eight mouse immunoglobulin isotype-specific rabbit monoclonal antibodies (IgG1κ, IgG1λ, IgG2aκ, IgG2aλ, IgG2bκ, IgG3κ, IgMκ, IgAκ, and IgAλ) was used to identify the isotypes of immunoglobulins produced by hybridomas according to the manufacturer's instructions. In brief, 50 μL of diluted isotype-specific rabbit anti-mouse purified monoclonal antibody combined with 50 μL of coating buffer was added into a 96-well plate and incubated overnight at 4 °C. Then, 100 μL/well of hybridoma culture supernatant and 100 μL/well of HRP-labeled rabbit anti-mouse Ig mAb solution were added and incubated as primary and secondary antibodies, respectively. After color development, the OD values at 450 nm were measured with an automatic microplate reader (TECAN, Männedorf, Switzerland).

### 2.6 Western blot analysis

All samples were mixed with an equal volume of loading buffer (0.125 M Tris-HCl (pH 6.8), 4.0% SDS, 20% glycerol, 10% 2-mercaptoethanol, and 0.004% bromophenol blue) and boiled at 95 °C for 5 min. Recombinant chTNF-ɑ was resolved on a 12% SDS-PAGE gel and electroblotted onto a nitrocellulose membrane (Thermo-Fisher Scientific, Waltham, USA). Subsequently, the membrane was blocked using 5% skim milk followed by washing with 1 x PBST and incubating with each mAb clone. After incubating with HRP-conjugated rabbit anti-mouse IgG secondary Ab (Thermo-Fisher Scientific, Waltham, USA), the bands were detected using a ncmECL Ultra (NCM Biotech, Suzhou, China) and visualized using the Tanon 5200 Multi imaging system (Tanon, Shanghai, China).

### 2.7 Indirect immunofluorescence

COS-7 cells were maintained in Dulbecco's modified Eagle's medium (DMEM) (Thermo-Fisher Scientific, Waltham, USA) supplemented with 10% heat-inactivated fetal bovine serum (Thermo-Fisher Scientific, Waltham, USA) and Penicillin-Streptomycin (Beyotime, Shanghai, China). For transient expression of chTNF-ɑ, COS-7 cells were transfected with 200 ng of pcDNA-chTNF-ɑ and pcDNA3.1 (+) vector (negative control), respectively, using Lipofectamine 2000 DNA Transfection Reagent (Thermo-Fisher Scientific, Waltham, USA) following the manufacturer's instructions. At 24 h after transfection, the cells were fixed with 70 μL of 4% paraformaldehyde for 15 min at room temperature, and after washing, the cells were incubated with 100 μL of 0.4% Triton X-100 for 10 min (Thermo-Fisher Scientific, Waltham, USA). After washing and blocking with 5% skim milk, the cells were reacted with the supernatant of each hybridoma (primary antibody) followed by fluorescein isothiocyanate (FITC)-conjugated goat anti-mouse IgG (H/L) (secondary antibody) (Thermo-Fisher Scientific, Waltham, USA). The reactivity of each mAb with chTNF-ɑ expressed in COS-7 cells was observed and photographed under an inverted fluorescence microscope (Nikon, Tokyo, Japan).

### 2.8 Construction and expression of chTNF-α polypeptide fragments

Three polypeptide fragments of chTNF-ɑ corresponding to amino acid residues (aa) 100–179, 159–233 and 195–285 were cloned using the codon-optimized chTNF-ɑ as template and inserted into pcDNA3.1 (+) vector to construct recombinant plasmid pcDNA-chTNF-ɑ-100-179, pcDNA-chTNF-ɑ-159-233 and pcDNA-chTNF-ɑ-195-285, respectively. Recombinant plasmids were transformed into *E. coli* DH5α competent cells (Takara, Kyoto-shi, Japan) and ultrapure plasmids were extracted. For transient transfection, 1 μg of each of the recombinant plasmids or empty pcDNA3.1 (+) vector was used to transfect COS-7 cells with Lipofectamine 2000 Transfection Reagent (Promega, Madison, USA). At 24 h after transfection, the reactivity of each mAb with TNF-ɑ polypeptide fragments was examined by indirect immunofluorescence assay (IFA) as above-mentioned protocol.

### 2.9 Dot-ELISA

A chTNF-α polypeptide of aa 167–196, which shared 33% homology with a neutralizing epitope of human TNF-α (Nagahira et al., 1995; Zhang et al., 2004), was synthesized by a commercial biological laboratory (HuaBio, Hangzhou, China) and subsequently conjugated to keyhole limpet hemocyanin (KLH). Dot-ELISA was performed to test the reactivity of each mAb with recombinant chTNF-α, KLH-conjugated chTNF-α polypeptide fragment (aa167-196), KLH and unrelated polypeptide, respectively. In brief, 3 μg of the concentrated protein was dotted onto the center of the nitrocellulose membrane, followed by drying at room temperature for 15 min. After drying, the unoccupied sites were blocked with 5% of skim milk for 1 h. The strips were then washed five times with PBST and incubated with the supernatant of each hybridoma (primary antibody) and HRP-conjugated goat anti-mouse IgG secondary Ab (Thermo-Fisher Scientific, Waltham, USA). Color was developed in the substrate solution and the reaction was stopped by distilled water. The results were observed with naked eyes.

### 2.10 Antigen-capture ELISA

An antigen-capture ELISA was developed to detect eukaryotic expressed chTNF-α protein produced by COS-7 cells and natural chTNF-α protein produced by chicken splenocytes upon stimulation with phorbol 12-myristate 13-acetate (PMA) (50 ng/mL) and ionomycin (500 ng/mL). In detail, the polyclonal and monoclonal antibodies were purified with Protein A/G agarose (Beyotime, Beijing, China), and the purity of antibodies were confirmed by SDS-PAGE. Subsequently, 96-well plates were coated with 100 μL of capture antibody (rabbit polyclonal antibodies, 5 μg/mL) overnight at 4 °C. After blocking and washing, 100 μL of diluted recombinant chTNF-α protein or lysate collected from pcDNA-chTNF-α transfected COS-7 cells or supernatant harvested from activated splenocytes was added and incubated for 2 h at 37 °C. After washing 5 times, each anti-chTNF-α monoclonal antibody was added and incubated for 1 h at room temperature. After washing again, HRP-conjugated goat anti-mouse IgG secondary Ab (Thermo-Fisher Scientific, Waltham, USA) was added and incubated for 1 h at room temperature. After final washing, color was developed in TMB solution and the OD values were measured at 450 nm. The mAb with lowest background was chosen as detection antibody for subsequent assay.

### 2.11 Statistical analysis

All statistical analyses were performed using the STATISTICA 7.0 software package (StatSoft, Oklahoma, USA). Independent Student's *t*-test was used to compare the values of OD_450_, positive/negative (P/N) ratios and relative expression levels of *chTNF-α*. All bar graphs in this study were constructed and analyzed with three or six replicates. All data were presented as mean ± SD and considered statistically significant when *P* ≤ 0.050.

## 3 Results

### 3.1 The quantification of LPS-induced natural chTNF-α mRNA

TNF-α is mainly produced by activated monocytes and macrophages in mammals. In order to detect the expression of chTNF-α by chicken macrophages, the chTNF-α mRNA expression was quantified by qPCR. As shown in Supplementary Figure 1, the expression level of chTNF-α mRNA in adherent macrophages from chicken PBMCs rapidly increased in 1 and 2 h after LPS treatment, compared to the control. However, at 8 h post-stimulation, there was no difference in the expression levels of chTNF-α mRNA between the two groups, suggesting chTNF-α is rapidly and early expressed upon activation.

### 3.2 Recombinant expression of chTNF-α in *E. coli* and COS-7 cells

In order to express recombinant chTNF-α protein**,** the extracellular domain of chTNF-α were cloned into prokaryotic and eukaryotic expression vectors. As shown in Figure 1B and 1D, a 558-bp of extracellular domain of chTNF-α was successfully inserted into pET45b and pcDNA3.1 vectors, and confirmed by restriction enzymatic digestion and sequencing. After IPTG induction, a 26-kD recombinant chTNF-α fused with 6xHis tag was expressed and purified with a concentration of 1.0 mg/L (Figure 1C). Similarly, the pcDNA-chTNF-α plasmid was transfected into COS-7 cells and a 22-kD recombinant chTNF-α was confirmed in the cell lysate by Western blot (Figure 1E).

### 3.3 Generation and characterization of anti-chTNF-α polyclonal and monoclonal antibodies

**Figure 2:**
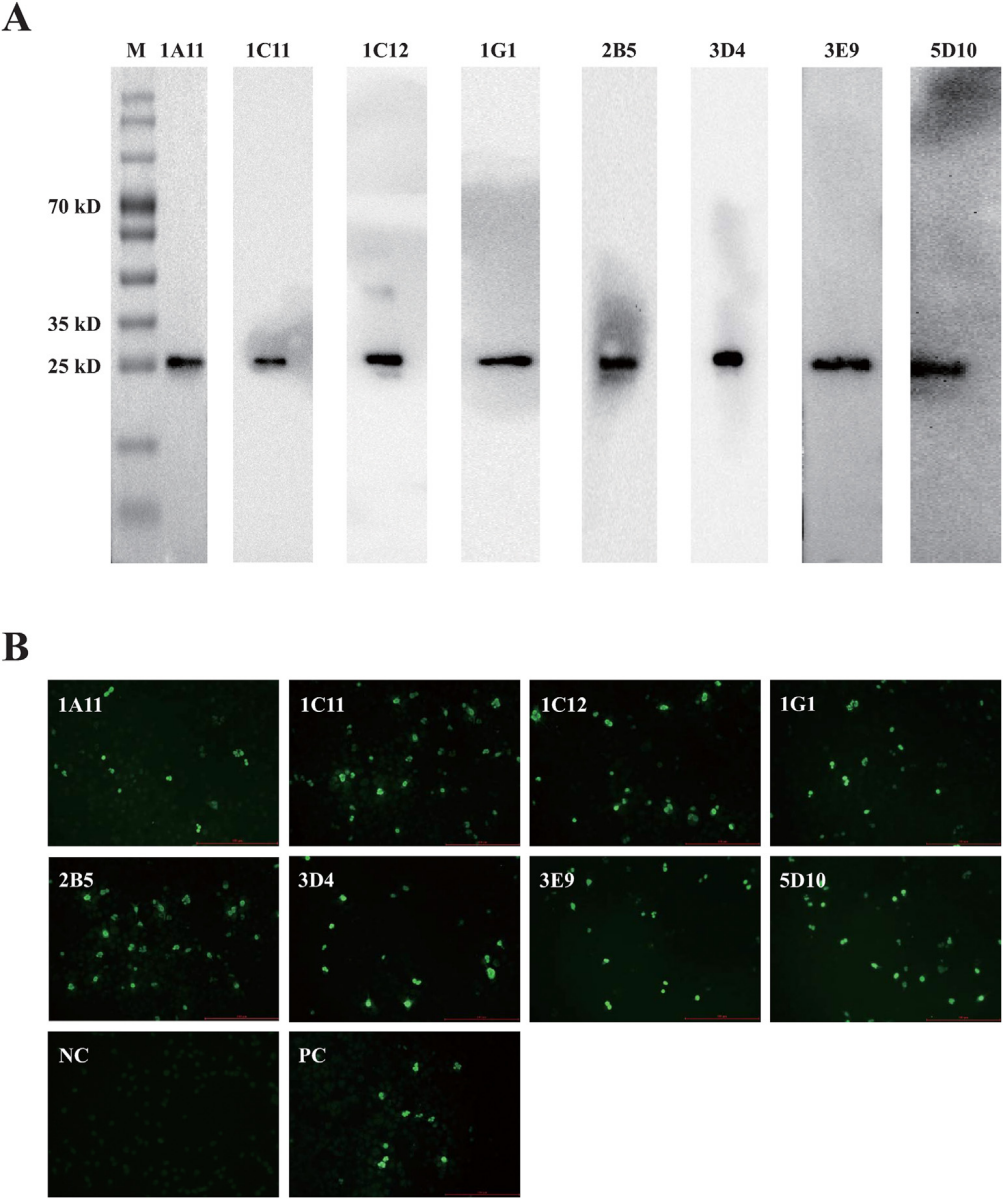
Identification of anti-chTNF-α monoclonal antibodies. The candidates of anti-chTNF-α monoclonal antibodies were screened using Western blot and indirect immunofluorescence with hybridoma culture supernatant. (A) Western blot was performed on prokaryotic recombinant chTNF-ɑ with eight clones (1A11, 1C11, 1C12, 1G1, 2B5, 3D4, 3E9 and 5D10) of hybridoma supernatant (primary antibody) and HRP-conjugated rabbit anti-mouse IgG (H + L) antibody (secondary antidody) (Thermo-Fisher Scientific, Waltham, USA). (B) The plasmids pcDNA-chTNF-ɑ and pcDNA3.1 (+) (served as NC) were transfected into COS-7 cells and incubated with eight clones (1A11, 1C11, 1C12, 1G1, 2B5, 3D4, 3E9 and 5D10) of hybridoma supernatant (primary antibody) or rabbit anti-chTNF-ɑ polyclonal antibody (positive control) and FITC-conjugated goat anti-mouse IgG (H/L) polyclonal antibody (Thermo-Fisher Scientific, Waltham, USA) or FITC-conjugated goat anti-rabbit IgG (H/L) polyclonal antibody (Beyotime, Shanghai, China) (secondary antibody). The uncropped images of Figure 2 (A) were referred in Supplementary Figure 6.

Using the purified chTNF-α as immunogen, eight clones of anti-chTNF-α mAbs were generated through hybridoma technology, which were named as 1A11, 1C11, 1C12, 1G1, 2B5, 3D4, 3E9 and 5D10, respectively (Table 1). Immunoglobulin isotyping showed that five clones of mAb (1A11, 1C12, 2B5, 3E9 and 5D10) were IgG1, whereas two clones (1C11 and 3D4) and one clone (1G1) belonged to IgG2a and IgM, respectively. The light chain of all the mAbs is κ chain (Table 1). The titers of these mAbs in the ascites ranged from 1:1,600 to 1:102,400. Western blot analysis showed that all these mAbs strongly reacted with the purified chTNF-α protein (Figure 2A). IFA showed that all these mAbs specifically recognized pcDNA-chTNF-α-transfected but not pcDNA3.1-transfected COS-7 cells (Figure 2B). These results suggested that all the mAbs specifically recognize prokaryotic and eukaryotic recombinant chTNF-α.

**Table 1:** Summary of the characteristics of monoclonal and polyclonal antibodies against chTNF-α identified by ELISA, Western blot and IFA.

Clone #	Isotype	Antibody titers	Western blot	IFA
1A11	IgG1, κ	1:1,600	++	+
1C11	IgG2a, κ	1:102,400	++	++
1C12	IgG1, κ	1:25,600	++	++
1G1	IgM, κ	1: 51,200	++	++
2B5	IgG1, κ	1: 6,400	++	++
3D4	IgG2a, κ	1:102,400	++	++
3E9	IgG1, κ	1:102,400	++	++
5D10	IgG1, κ	1:12,800	++	++
pAb		1:51,200	++	++

Similarly, the ELISA results showed that the titer of rabbit anti-chTNF-α pAb was 1:51,200 (Table 1). This pAb could specifically react with recombinant chTNF-α expressed in pcDNA-chTNF-α-transfected COS-7 cells by IFA (Supplementary Figure 2).

### 3.4 Epitope mapping

**Figure 3:**
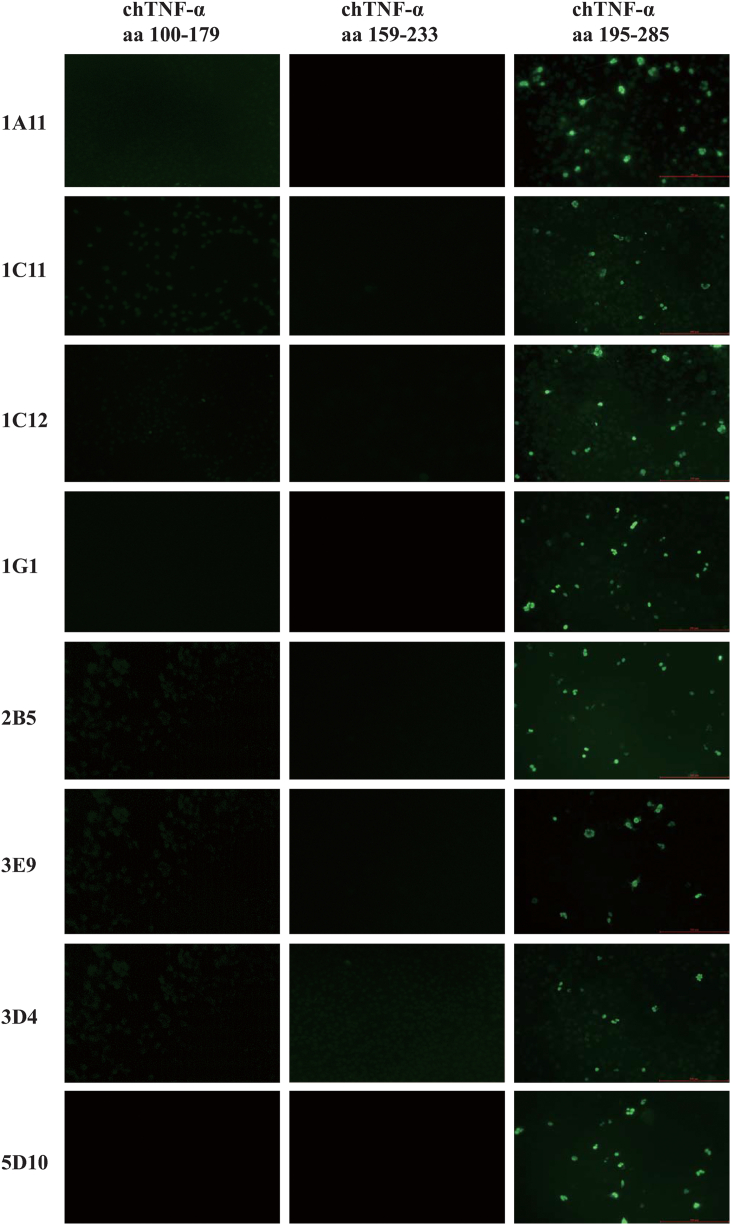
Epitope mapping of anti-chTNF-α monoclonal antibodies. Three chTNF-α polypeptide fragments (aa 100–179, 159–233 and 195–285) were cloned and ligated into plasmid pcDNA3.1 (+). Indirect immunofluorescence assay was performed on these three recombinant plasmids with eight candidates of monoclonal antibodies (1A11, 1C11, 1C12, 1G1, 2B5, 3D4, 3E9 and 5D10) (primary antibody) and FITC-conjugated goat anti-mouse IgG (H/L) polyclonal antibody (Thermo-Fisher Scientific, Waltham, USA) (secondary antibody).

In order to identify the antigenic region or epitopes recognized by these mAbs, we constructed three plasmids expressing the polypeptide of chTNF-α aa 100–179, 159–233 and 195–285, respectively (Supplementary Figure 3) and tested the reactivity of each mAb with these fragments expressed in COS-7 cells after transfection. IFA showed that all the eight mAbs recognized the epitope region aa 195–285 of chTNF-α but not the other regions (Figure 3). As the region of aa 167–196 of human TNF-α was shown to be a neutralizing epitope (Hehlgans and Pfeffer, 2005; Pfeffer, 2003), we performed dot-ELISA to identify whether any mAb to chTNF-ɑ recognizes the corresponding region of chTNF-ɑ. The results showed that none of those mAbs reacted with KLH-conjugated chTNF-α polypeptide (aa 167–196), KLH and unrelated polypeptide except recombinant chTNF-ɑ (Supplementary Figure 4).

### 3.5 Selection of detection antibody

**Figure 4:**
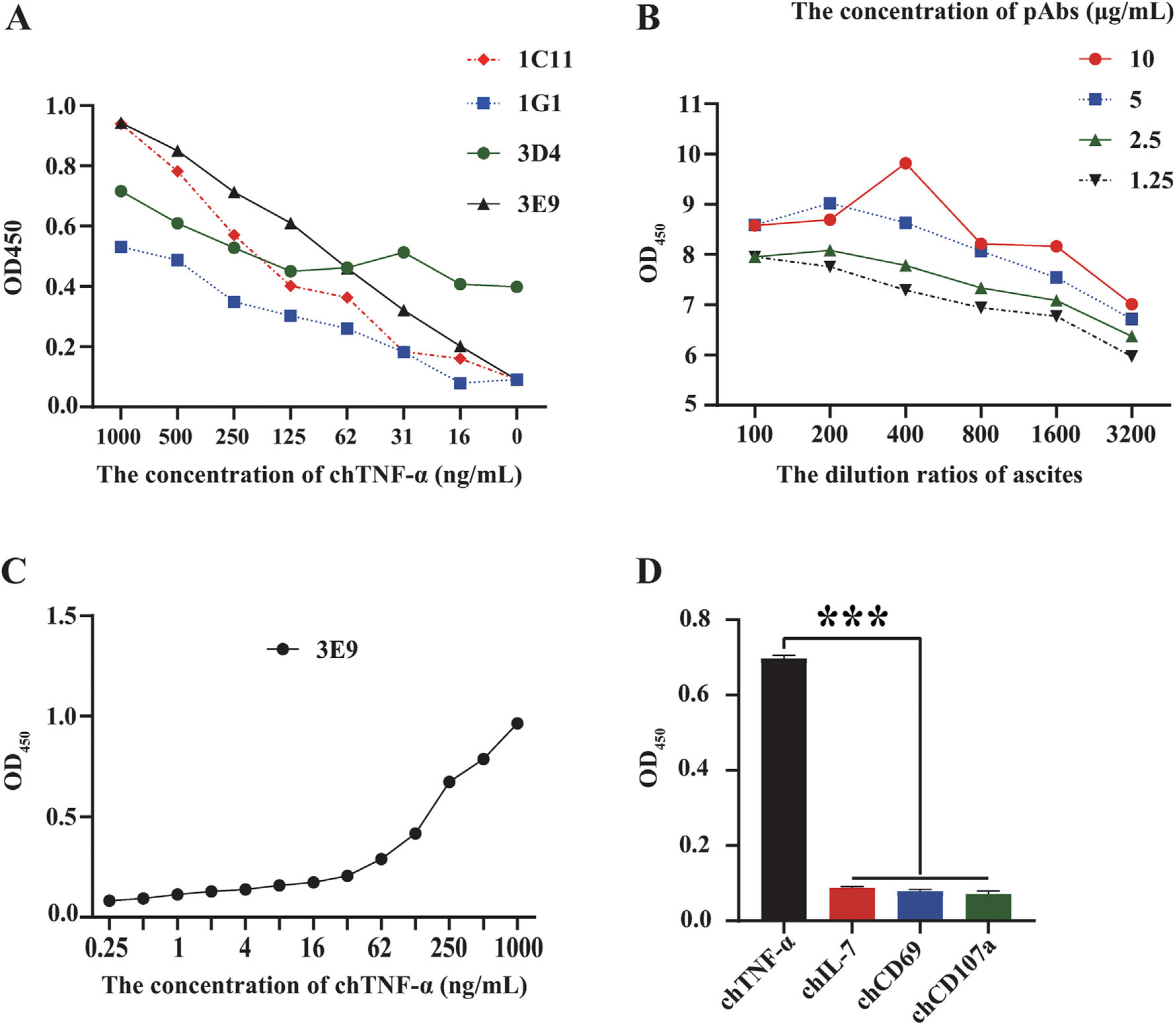
Sensitivity and specificity of the antigen-capture ELISA. (A) Antigen-capture ELISA was performed with four clones [1C11 (in red), 1G1 (in blue), 3D4 (in green) and 3E9 (in black)] of anti-chTNF-α monoclonal antibodies (three replicates). (B) Antigen-capture ELISA was performed to determine the optimal concentrations [10 (in red), 5 (in blue), 2.5 (in green) and 1.25 (in black), μg/mL] of antigen-capture and detection antibodies (three replicates). (C) The sensitivity of this antigen-capture ELISA was evaluated with different concentrations of recombinant chTNF-α (three replicates). (D) The specificity of this antigen-capture ELISA was evaluated with chTNF-α (in black), chIL-7 (in red), chCD69 (in blue) and chCD107a (in green) (six replicates).

In this study, rabbit anti-chTNF-α pAb was used as antigen-capture antibody, and mouse anti-chTNF-α monoclonal antibodies were employed as detection antibody. By pairing with the pAb in an ELISA assay, four clones of mAbs (1C11, 1G1, 3D4 and 3E9) with strong reactivity in IFA were chosen and evaluated for their sensitivity and specificity in the detection of recombinant chTNF-α protein. The results showed that mAb 3E9 has highest sensitivity and lowest background threshold (Figure 4A). As shown in Figure 4B, by checkboard titration, the optimal coating concentrations of the pAb was determined to be 10 μg/mL, while the optimal dilution of detection antibody 3E9 was 1:400, which gave rise to highest P/N ratio (9.82).

### 3.6 Sensitivity and specificity of the antigen-capture ELISA

To determine the sensitivity of the designed ELISA, recombinant chTNF-α was used as standard antigen and diluted serially from 1000 ng/mL to 0.25 ng/mL. As depicted in Figure 4C, the detection limitation of this antigen-capture ELISA is 1 ng/mL based on the cut-off values (P/N ≥ 2.1).

As for the specificity, this assay was employed to detect three irrelevant Trx-fused antigens (chIL-7, chCD69 and chCD107a) that were expressed in-house. The results showed that this assay did not have cross-reactivity with those irrelevant antigens tested. The OD_450_ values of three antigens were close to that of negative control (PBS), and significantly lower than that of chTNF-α, indicating that this antigen-capture ELISA is specific for the detection of chTNF-α (Figure 4D).

### 3.7 Detection of natural chTNF-α by the antigen-capture ELISA

**Figure 5:**
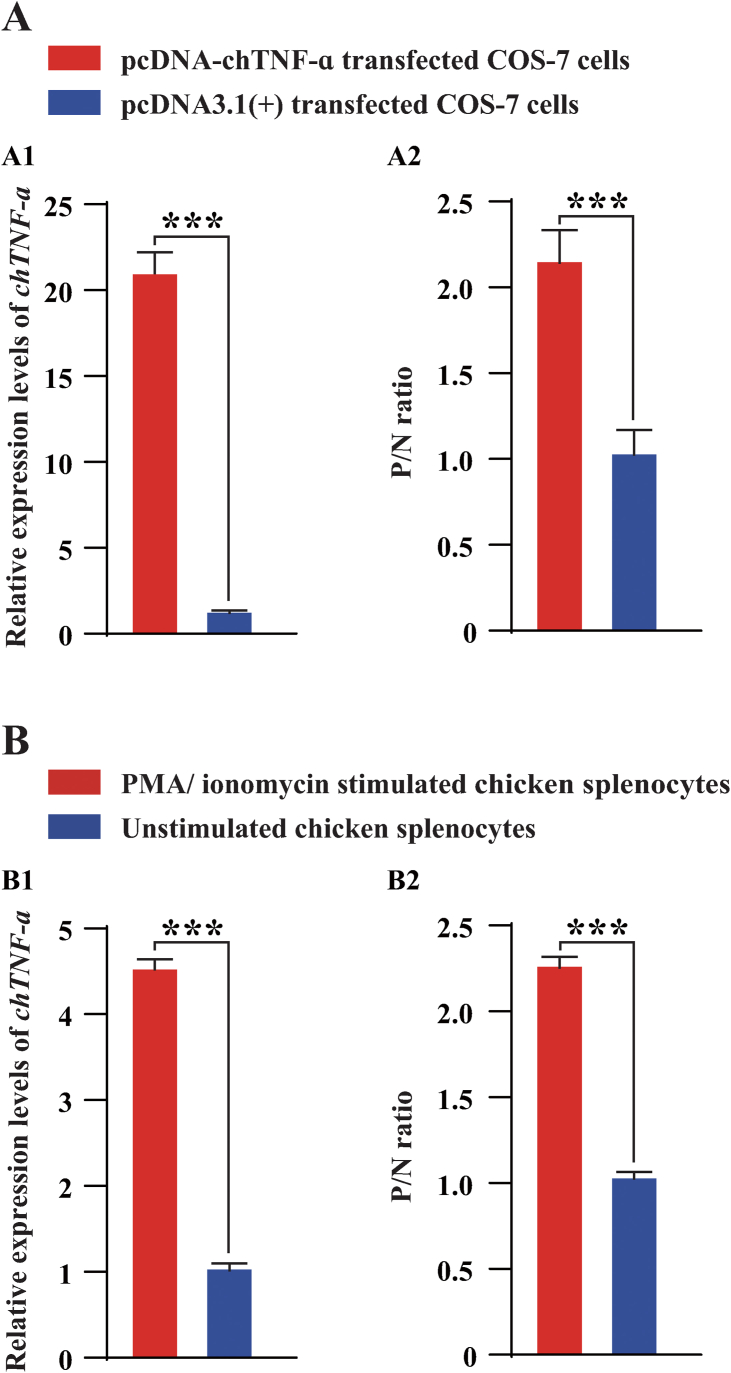
Comparison of the expression of chTNF-α in COS-7 cells (A) transfected with pcDNA-chTNF-α (in red) and pcDNA-3.1 (+) (in blue) and in chicken splenocytes (B) with (in red) and without (in blue) PMA/ionomycin stimulation. The expression of chTNF-α was detected using RT-qPCR (A1 and B1) and the antigen-capture ELISA (A2 and B2) designed in this study (six replicates). Data were shown as mean ± SD and the symbol (∗∗∗) indicated *P* ≤ 0.001.

To evaluate the efficacy of this antigen-capture ELISA in detecting natural chTNF-α, COS-7 cells were transfected with pcDNA-chTNF-α and chicken splenocytes were stimulated with PMA and ionomycin for 6 h and the lysate/supernatant was collected for the detection of natural chTNF-α. The results showed that significant higher expressions (*P* ≤ 0.001) of chTNF-α mRNA was quantified by RT-qPCR in the cells after transfection/activation (Figure 5A1 and 5B1), which was consistent with the expression changes (*P* ≤ 0.001) of chTNF-αin the lysate/supernatant detected by this antigen-capture ELISA (Figure 5A2 and 5B2).

## 4 Discussion

To date, a few of avian whole genome sequences have been released into public database (Huang et al., 2013; International Chicken Genome Sequencing, 2004; Lee et al., 2020; Shapiro et al., 2013). However, due to high GC content and multiple long GC-rich stretches, there are still hundreds of genes missing in birds that are present in most of other vertebrate lineages (Hron et al., 2015). Unfortunately, it is unknown why birds display significant differences in these specific genes from other vertebrate, but one most plausible explanation is the GC-biased gene conversion (Rousselle et al., 2019). Although chicken lipopolysaccharide-induced TNF-α factor (LITAF) has been cloned and characterized in 2006 (Hong et al., 2006), authentic chTNF-α gene, which is unrelated to LITAF, has not been identified until very recently (Rohde et al., 2018). The discovery of chTNF-α gene has led to the requirement of further investigation of its biological functions, which need immunological reagents for its detection at first. In this study, through recombinant expression of the extracellular domain of chTNF-α and hybridoma technology, we generated and characterized chTNF-α-specific mAbs and rabbit pAb. These antibodies specifically recognized eukaryotically-expressed chTNF-ɑ and can be used to detect natural chTNF-ɑ in an antigen-capture ELISA assay, providing important tools for the immunological characterization of chTNF-ɑ in the future.

Although the full-length chTNF-α gene was cloned and released in the Genbank of National Center for Biotechnology Information (Genbank access # MF000729), it was very difficult to clone the full-length gene in our hands probably due to the high GC content or long GC-rich stretches in the gene (Hron et al., 2015). Initially, we tried to clone the complete coding sequence of chTNF-α using five pairs of specific primers by overlapping PCR, it turned out to be very hard to fuse the fragments at 200-300nt of the chTNF-α gene. Even though these five fragments had been successfully fused into a full-length gene and cloned into the T-vector, the recombinant plasmid somehow could not be completely sequenced and confirmed. Therefore, we had to choose the extracellular domain of chTNF-α for recombinant expression. As for the eukaryotic expression of chTNF-α, we had tried to insert the extracellular domain of chTNF-α into pCI-neo and pEGFP vectors for its expression in the transfected COS-7 and DF-1 cells but failed. Eventually, the recombinant chTNF-α (codon optimized) was expressed in the pcDNA3.1-chTNF-α-transfected COS-7 and DF-1 cells.

TNF-α is mainly produced by monocytes and macrophages, which can be easily detected in mammals, and plays important roles in a variety of inflammatory responses and immunopathogenesis. The biological activities and functions of TNF-α have been described in different pathological processes in humans and mammals, including neoplastic diseases (Balkwill, 2009), mental disorders (Ma et al., 2016) and inflammation-mediated biological defense functions (Kushibiki, 2011). In contrast, the study of avian TNF-α is still in its infancy. The availability of chTNF-α-specific immunological tools would facilitate the studies of the biological function of chTNF-α as well as its application in poultry diseases. At present, commercial ELISA kits have been developed and applied to detect TNF-α in humans, mice, rats and other mammals (Chen et al., 2020; Han et al., 2019). However, due to the low homology of TNF-α genes between avian and mammalian species, they cannot be used for the detection of chTNF-α. In this study, a total of eight clones of anti-chTNF-α mAbs were generated. Epitope mapping showed that all these mAbs interacted with aa 195–285 peptide of chTNF-α, but not with aa 159–233. As there is an overlapping region existing between these two peptides, it can be deduced that the mAbs obtained in this study probably all recognized the region of aa 234–285. In addition, we developed an antigen-capture ELISA assay for the detection of natural chTNF-α by pairing mAb 3E9 and the rabbit pAb. This assay showed limited detection sensitivity of natural chTNF-α at a minimal concentration of 1 ng/mL (Figure 4), which is 30 and 125 times less sensitive than the commercial TNF-α ELISA kits for mouse (31.3–2,000 pg/mL, BMS607-3, ThermoFisher) and human (7.8–500 pg/mL, CSB-E04740h, CUSABIO), respectively. As those commercial ELISA kits are coated with mAbs against mouse or human TNF-α, it is unclear whether the pAb used as a capture antibody in this study affects the sensitivity of this assay. Further optimization of this assay may be needed. It is worth noting that the sensitivity of the ELISA developed in this study is comparable to that of a very recent study (0.5 ng/mL), in which two clones of mAbs were used (Lu et al., 2022). The use of rabbit pAb could effectively reduce the cost. In addition, in this study, the antigenic epitopes of these mAbs were identified to recognize a region of aa 195–285 of chTNF-ɑ.

Overall, we generated chTNF-α-specific mAbs and rabbit pAb and developed an antigen-capture ELISA for the detection of natural chTNF-α. Our work provides important immunological tools for the study of the functions of the newly-identified chTNF-ɑ and would promote the advance of avian immunology.

## Declarations

### Author contribution statement

Yi Yang and Shaobin Shang: Conceived and designed the experiments; Analyzed and interpreted the data; Contributed reagents, materials, analysis tools or data; Wrote the paper.

Yining Meng, Lina Chen, Maoli Dong, Huining Zhang, Ji Wu, Shuangjiang He, Yunfei Tian and Zaicheng Gong: Performed the experiments.

Xiaoli Hao: Analyzed and interpreted the data.

### Funding statement

Xiaoli Hao was supported by National Natural Science Foundation of China (32002293).

Yi Yang was supported by Basic Research Program of Jiangsu Province (BK20190881), Innovation and Entrepreneurship Program of Jiangsu Province ((2019) 30150), “Qing Lan Project” of Yangzhou University, China (YDRS (2020) 16), the “High-end talent support program” of Yangzhou University, China (YDRS (2022) 5), Priority Academic Program Development of Jiangsu Higher Education Institutions (PAPD).

Shaobin Shang was supported by Priority Academic Program Development of Jiangsu Higher Education Institutions (PAPD).

### Data availability statement

Data included in article/supp. material/referenced in article.

### Declaration of interest’s statement

The authors declare no conflict of interest.

### Additional information

Supplementary content related to this article has been published online at [URL].
