# Supplementary material for: Development of specific monoclonal antibodies for the detection of natural chicken tumor necrosis factor-alpha
Source: Heliyon. 2022 Dec 21;8(12):e12446. doi: 10.1016/j.heliyon.2022.e12446 (PMC9803707; doi:10.1016/j.heliyon.2022.e12446)
Supplement: Supplementary Figures 1-6 [file mmc1.docx]

Supplementary Material


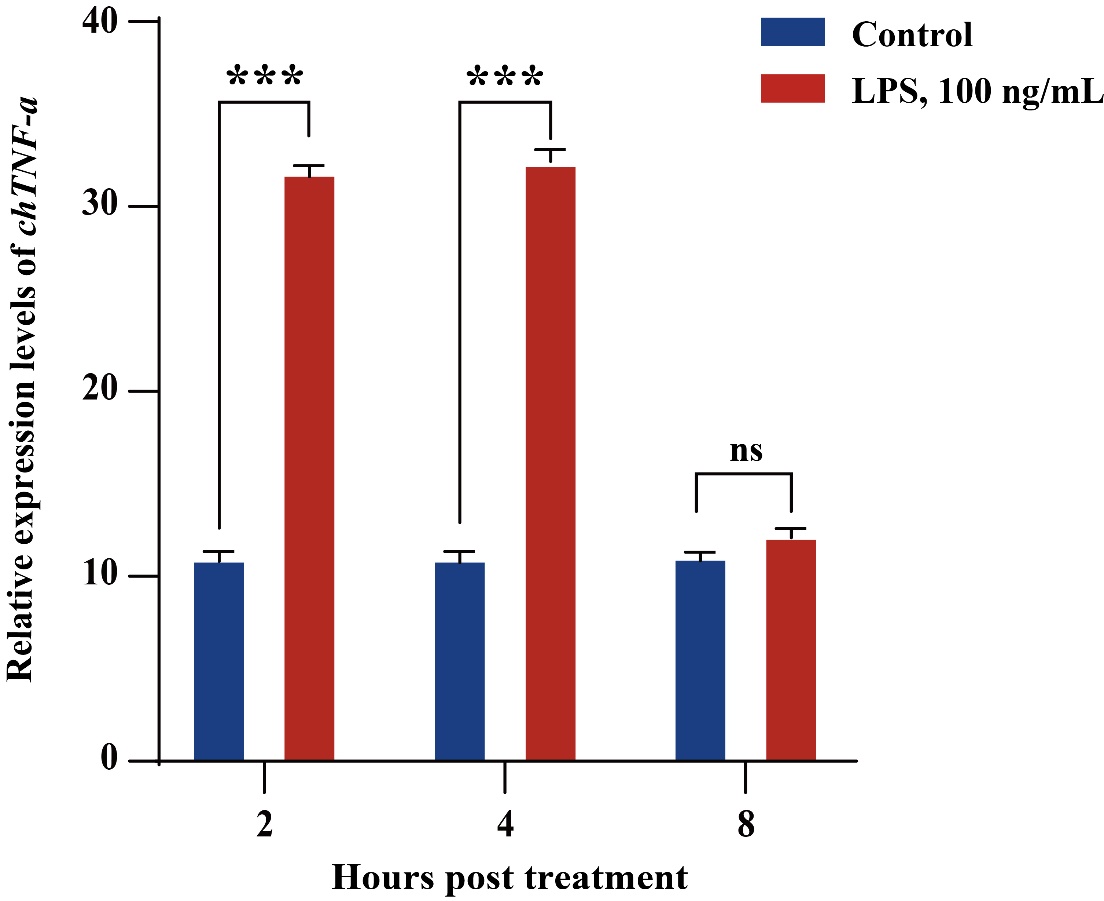


**Supplementary Figure 1.** The expression of natural chTNF-α in chicken peripheral blood macrophages stimulated by LPS. The expression levels of natural chTNF-α in LPS stimulated (in red) and control (in blue) peripheral blood macrophages were compared and analyzed by student’s *t* test. Data were shown as mean ± SD and the symbols (** and ns) indicated *P* ≤ 0.001 and *P* > 0.050, respectively.

**
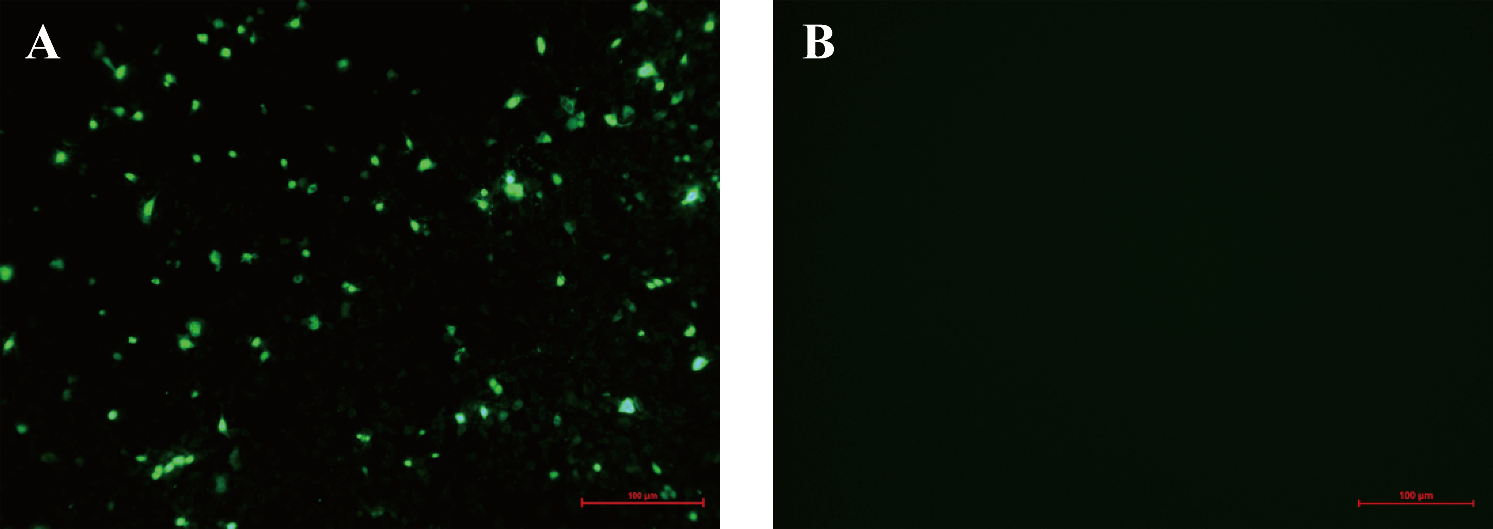
**

**Supplementary Figure 2.** Identification of rabbit anti-chTNF-α polyclonal antibodies using indirect immunofluorescence. The rabbit anti-chTNF-α polyclonal antibodies were reacted with the COS-7 cells transfected with pcDNA-chTNF-ɑ **(A)** and pcDNA3.1(+) **(B)**, respectively.


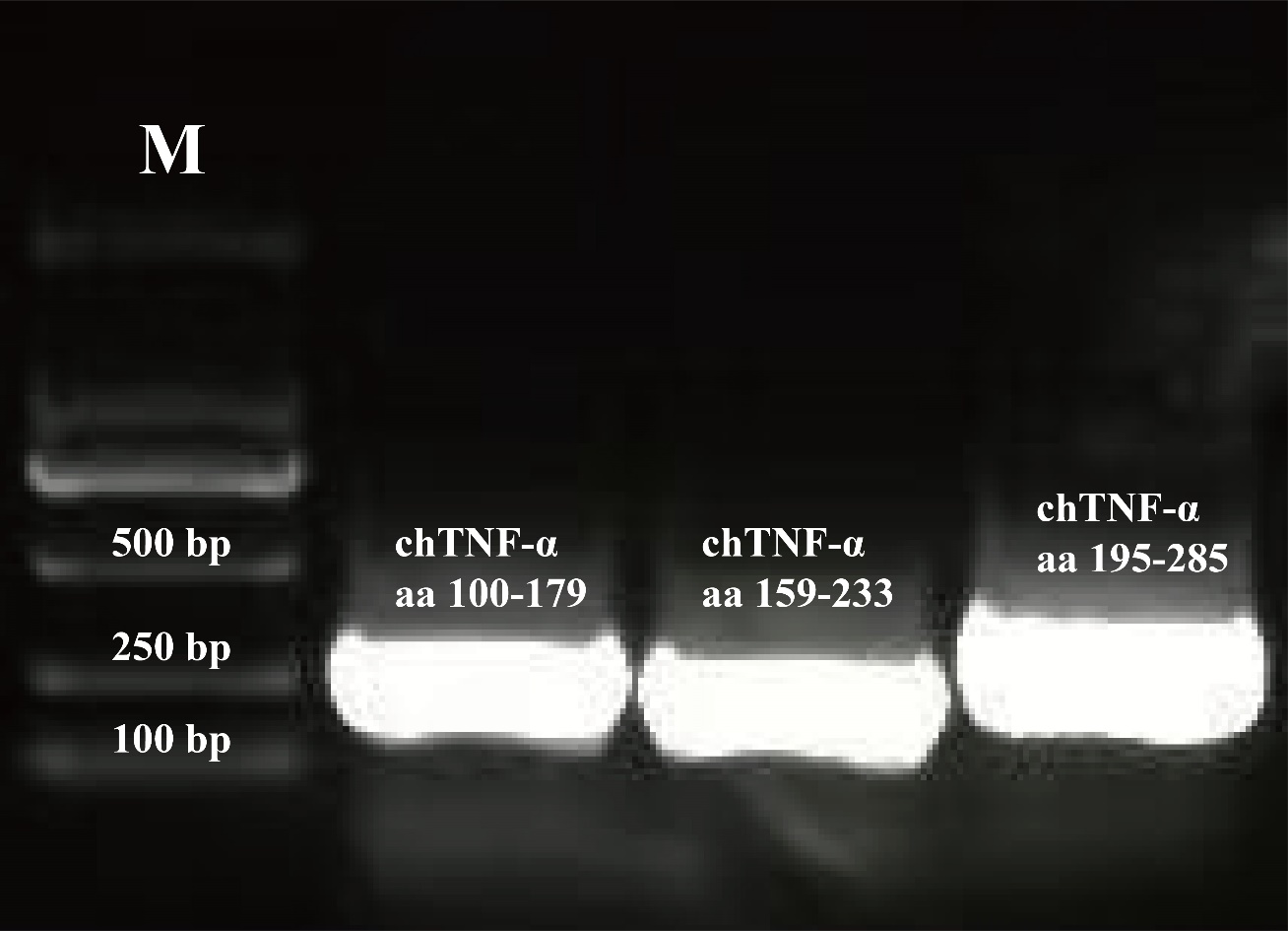


**Supplementary Figure 3.** The amplification and identification of three chTNF-α polypeptides (chTNF-α aa 100-179, 159-233 and 195-285).


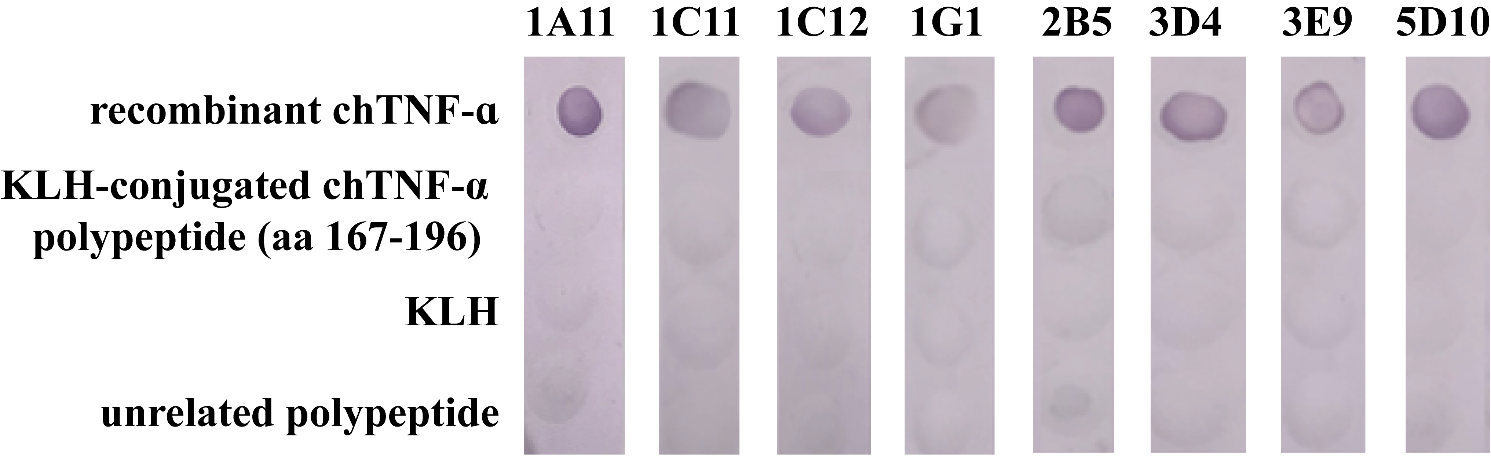


**Supplementary Figure 4.** Eight clones of hybridoma supernatant (1A11, 1C11, 1C12, 1G1, 2B5, 3D4, 3E9 and 5D10) were reacted with recombinant chTNF-ɑ and KLH-conjugated chTNF-α polypeptide (aa 167-196) with dot-ELISA.


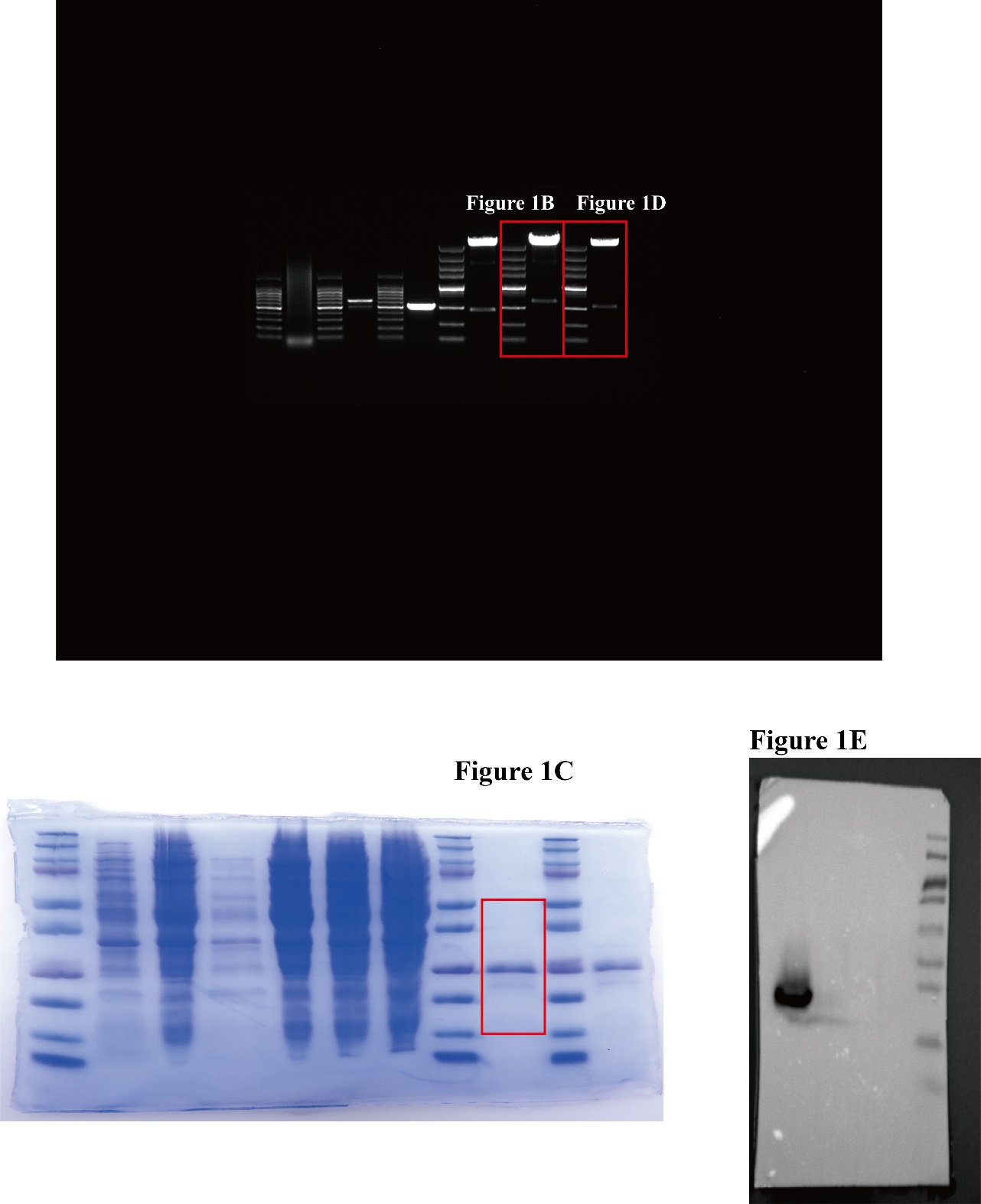


**Supplementary Figure 5.** Uncropped images of gels and blots in Figure 1 (B-E).


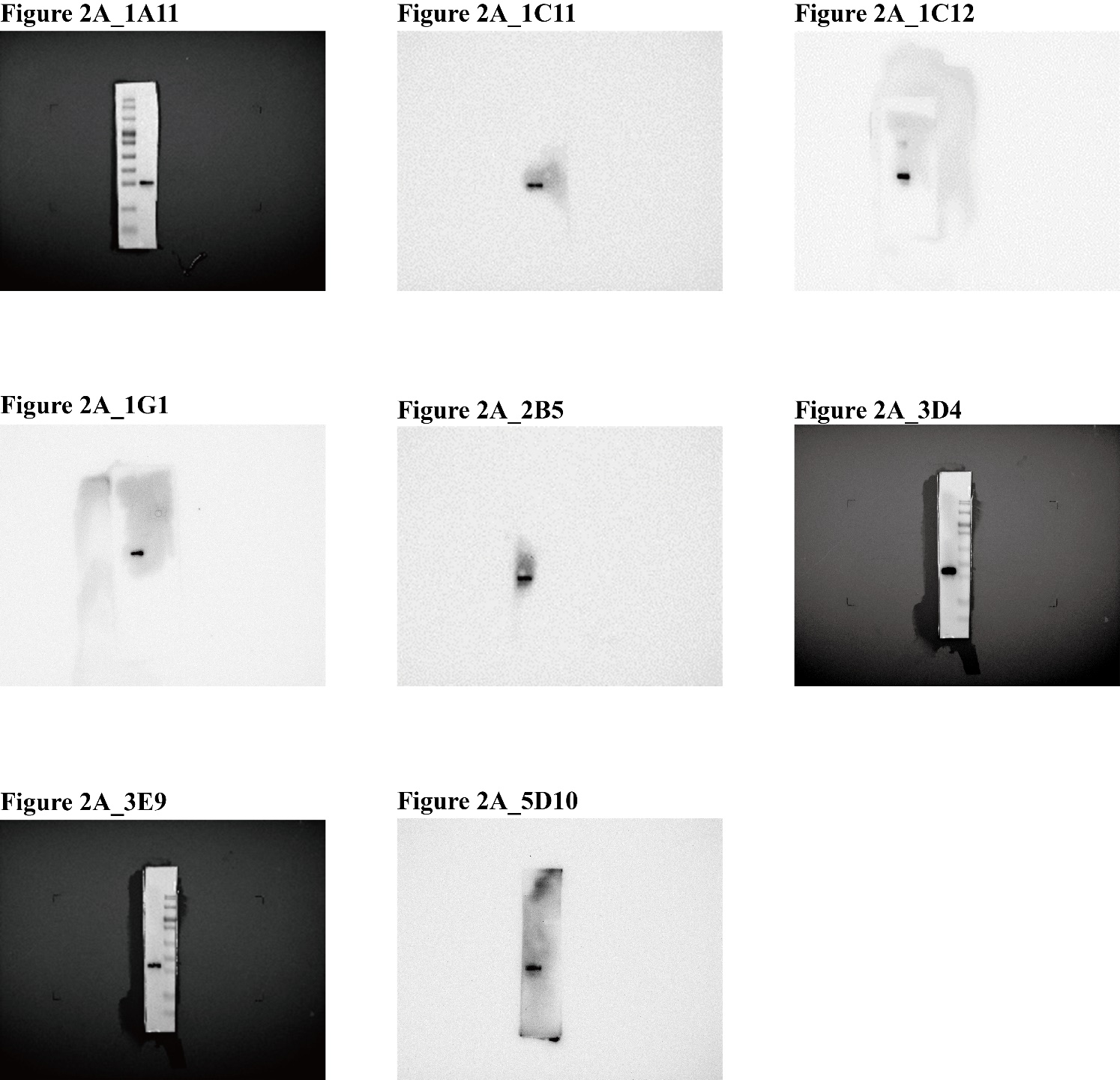


**Supplementary Figure 6.** Uncropped images of blots in Figure 2 (A).
